# Supplementary figures and images for: Periplaneta americana Extract Protects Glutamate-Induced Nerve Cell Damage by Inhibiting N-Methyl-D-Aspartate Receptor Activation
Source: Biology (Basel). 2025 Feb 13;14(2):193. doi: 10.3390/biology14020193 (PMC11851986; doi:10.3390/biology14020193)

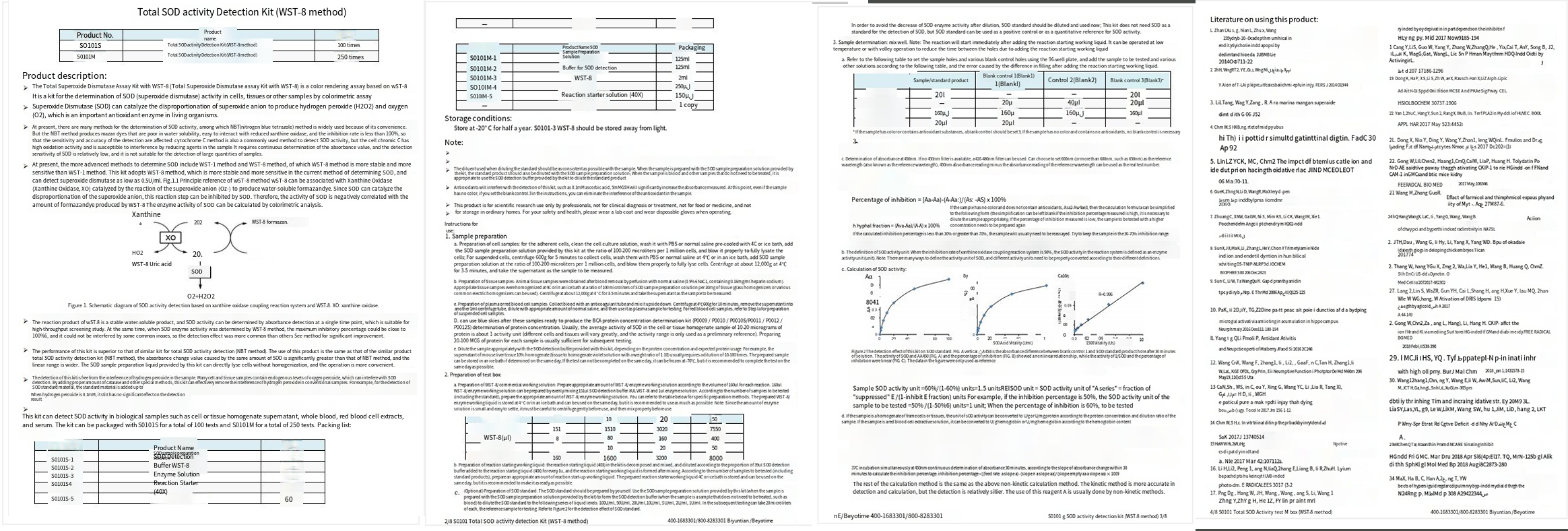

Supplement: Supplementary file 1 [file biology-14-00193-s001.zip › Figure S1, S0101 SOD test kit(WST-8).tif]

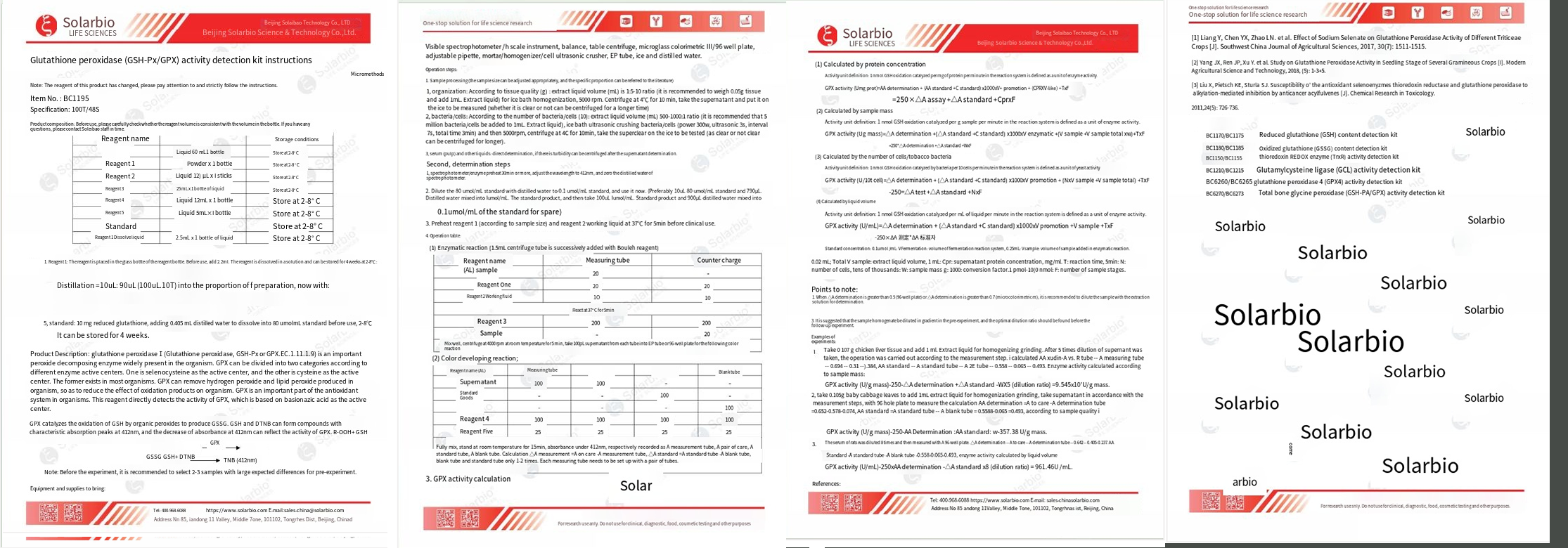

Supplement: Supplementary file 1 [file biology-14-00193-s001.zip › Figure S2, BC1195 GSH-Px TEST KIT.tif]

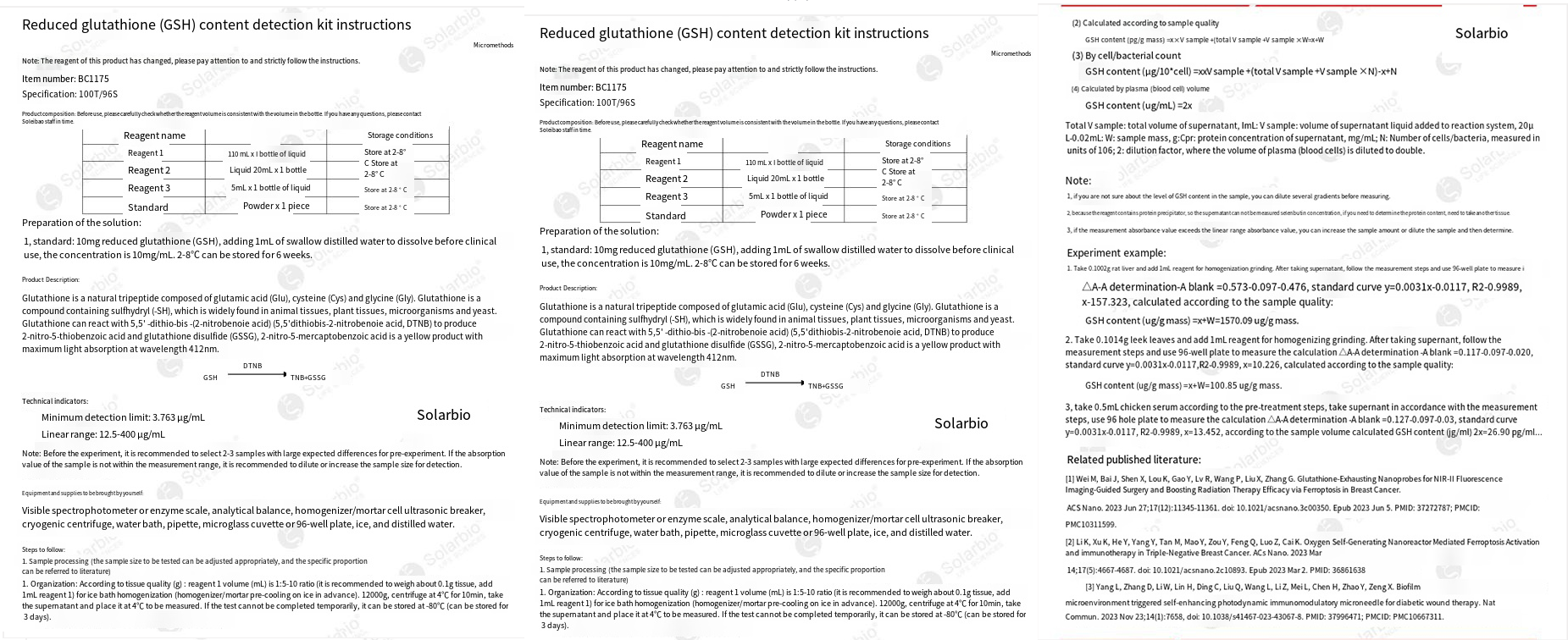

Supplement: Supplementary file 1 [file biology-14-00193-s001.zip › Figure S3, BC1175 GSH test kit.tif]

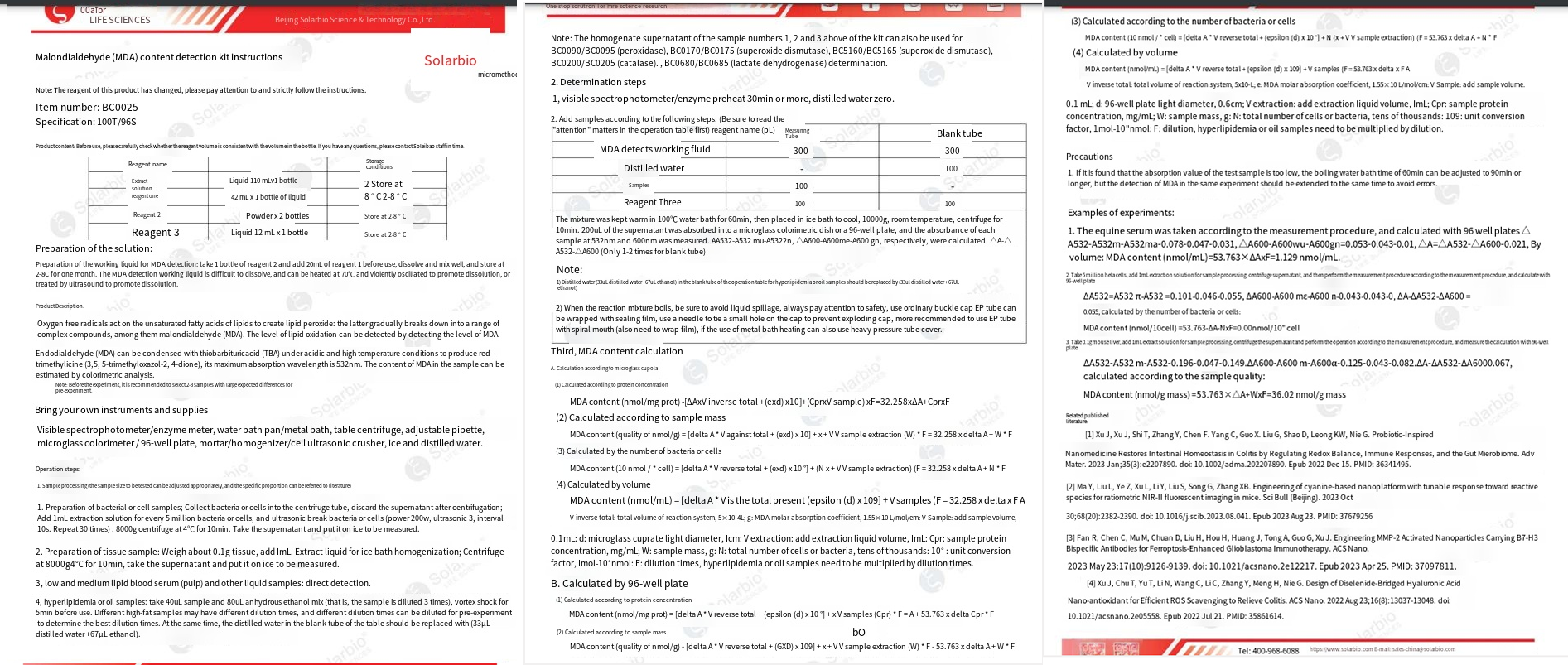

Supplement: Supplementary file 1 [file biology-14-00193-s001.zip › Figure S4, BC0025 MDA test kit.tif]

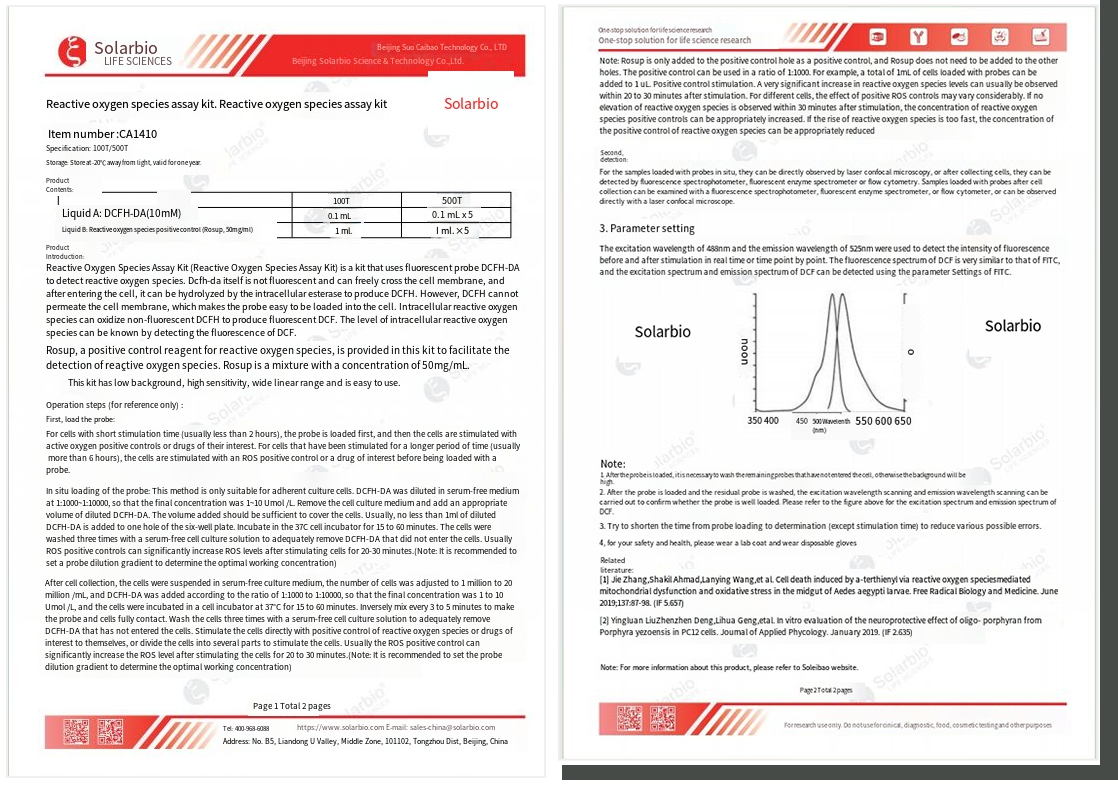

Supplement: Supplementary file 1 [file biology-14-00193-s001.zip › Figure S5, ROS test kit.tif]

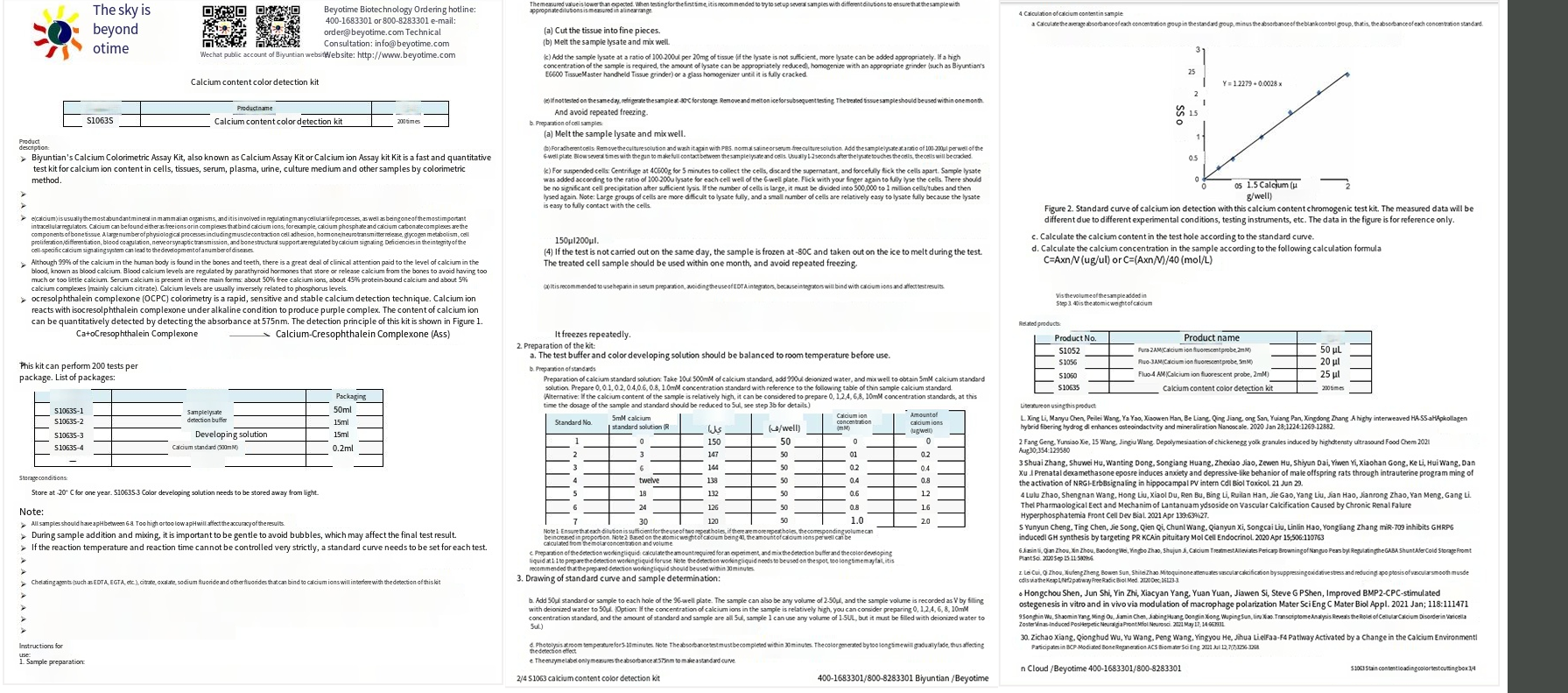

Supplement: Supplementary file 1 [file biology-14-00193-s001.zip › Figure S6, S1063 Calcium content color detection kit.tif]

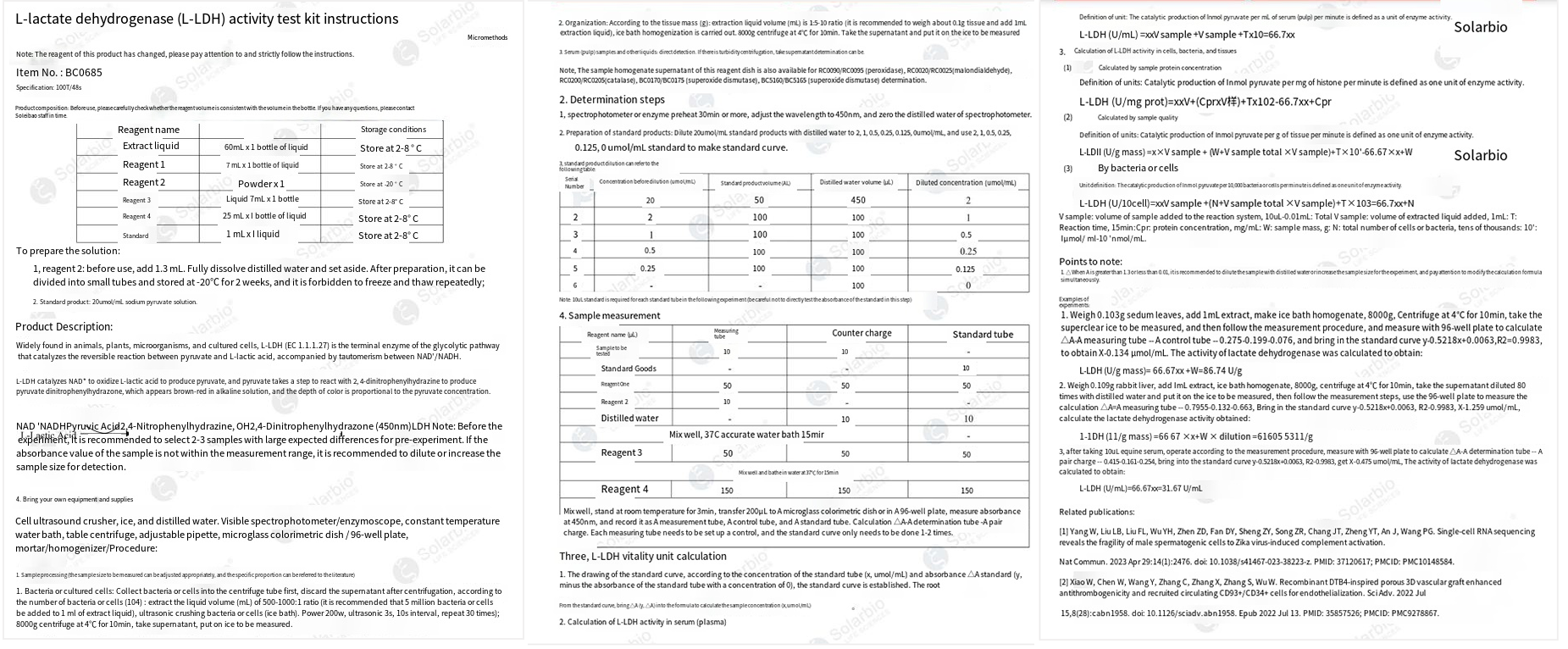

Supplement: Supplementary file 1 [file biology-14-00193-s001.zip › Figure S7, BC0685 LDH TEST KIT.tif]

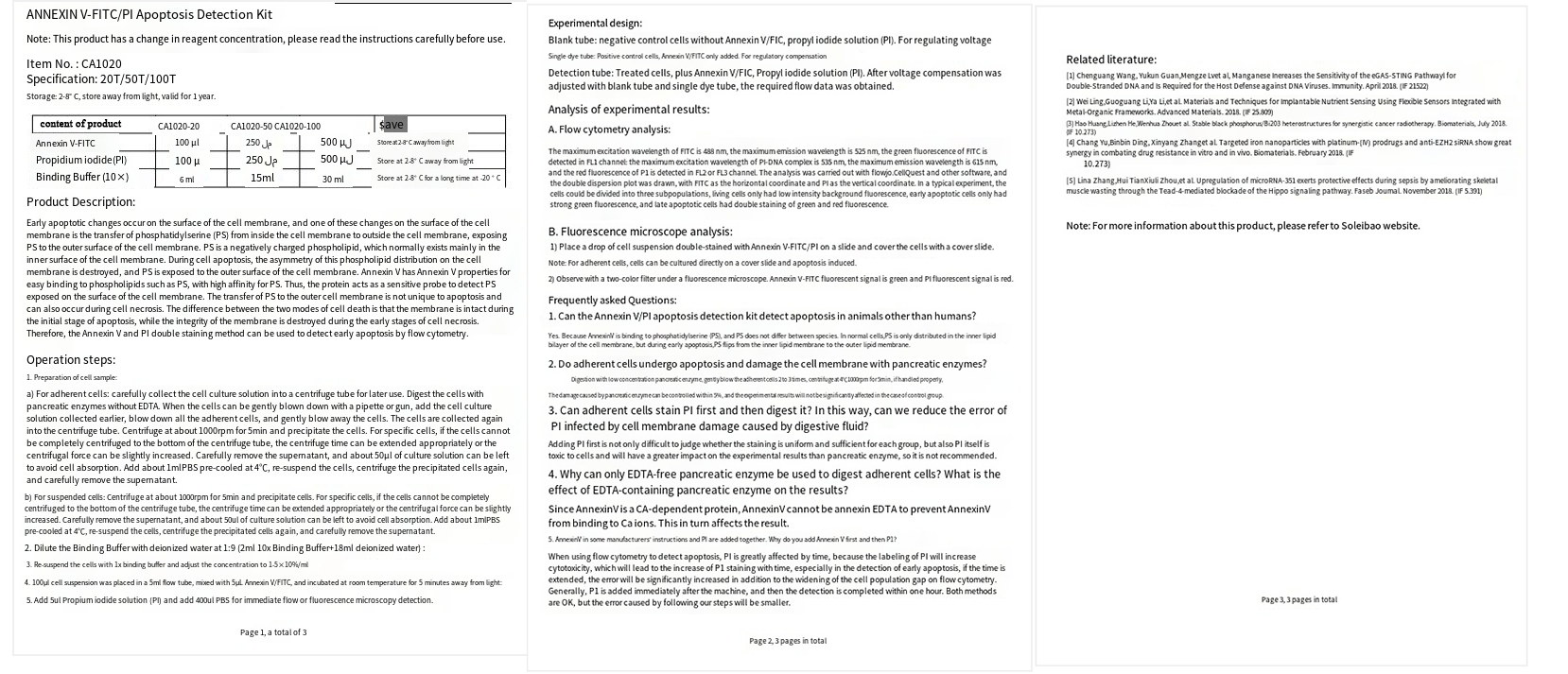

Supplement: Supplementary file 1 [file biology-14-00193-s001.zip › Figure S8, Annexin V FITC-PE test kit.tif]

|          |                                                                                      |
|----------|--------------------------------------------------------------------------------------|
| Bax      | 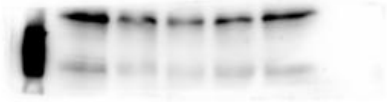   |
| Bcl-2    | 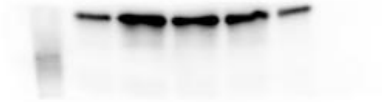   |
| Caspase3 | 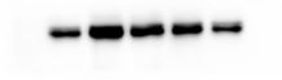 |
| Cytc     | 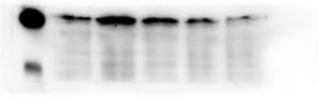 |

NMDAR1

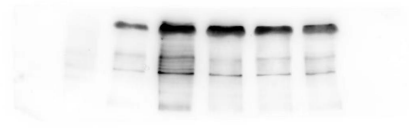

GAPDH

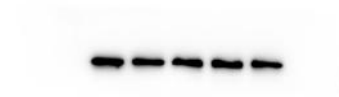

Supplement: Supplementary file 1 [file biology-14-00193-s001.zip › Western Blot.pdf]
